# Genome-wide association study presents insights into the genetic architecture of drought tolerance in maize seedlings under field water-deficit conditions

**DOI:** 10.3389/fpls.2023.1165582

**Published:** 2023-05-08

**Authors:** Shan Chen, Dongdong Dang, Yubo Liu, Shuwen Ji, Hongjian Zheng, Chenghao Zhao, Xiaomei Dong, Cong Li, Yuan Guan, Ao Zhang, Yanye Ruan

**Affiliations:** ^1^ Shenyang City Key Laboratory of Maize Genomic Selection Breeding, College of Bioscience and Biotechnology, Shenyang Agricultural University, Shenyang, Liaoning, China; ^2^ CIMMYT-China Specialty Maize Research Center, Crop Breeding and Cultivation Research Institute, Shang-hai Academy of Agricultural Sciences, Shanghai, China; ^3^ International Maize and Wheat Improvement Center (CIMMYT), Texcoco, Mexico; ^4^ Dandong Academy of Agricultural Sciences, Fengcheng, Liaoning, China

**Keywords:** maize (*Zea mays* L.), genome-wide association study, seedling stage, field drought tolerance, SNPs

## Abstract

**Introduction:**

Drought stress is one of the most serious abiotic stresses leading to crop yield reduction. Due to the wide range of planting areas, the production of maize is particularly affected by global drought stress. The cultivation of drought-resistant maize varieties can achieve relatively high, stable yield in arid and semi-arid zones and in the erratic rainfall or occasional drought areas. Therefore, to a great degree, the adverse impact of drought on maize yield can be mitigated by developing drought-resistant or -tolerant varieties. However, the efficacy of traditional breeding solely relying on phenotypic selection is not adequate for the need of maize drought-resistant varieties. Revealing the genetic basis enables to guide the genetic improvement of maize drought tolerance.

**Methods:**

We utilized a maize association panel of 379 inbred lines with tropical, subtropical and temperate backgrounds to analyze the genetic structure of maize drought tolerance at seedling stage. We obtained the high quality 7837 SNPs from DArT's and 91,003 SNPs from GBS, and a resultant combination of 97,862 SNPs of GBS with DArT's. The maize population presented the lower her-itabilities of the seedling emergence rate (ER), seedling plant height (SPH) and grain yield (GY) under field drought conditions.

**Results:**

GWAS analysis by MLM and BLINK models with the phenotypic data and 97862 SNPs revealed 15 variants that were significantly independent related to drought-resistant traits at the seedling stage above the threshold of P < 1.02 × 10-5. We found 15 candidate genes for drought resistance at the seedling stage that may involve in (1) metabolism (*Zm00001d012176*, *Zm00001d012101*, *Zm00001d009488*); (2) programmed cell death (*Zm00001d053952*); (3) transcriptional regulation (*Zm00001d037771*, *Zm00001d053859*, *Zm00001d031861*, *Zm00001d038930*, *Zm00001d049400*, *Zm00001d045128* and *Zm00001d043036*); (4) autophagy (*Zm00001d028417*); and (5) cell growth and development (*Zm00001d017495*). The most of them in B73 maize line were shown to change the expression pattern in response to drought stress. These results provide useful information for understanding the genetic basis of drought stress tolerance of maize at seedling stage.

## 1 Introduction

Maize (*Zea mays* L.) is a critical source of food, feed, and energy because of its high yield potential. Poor harvest of maize caused by environmental stresses will have a tremendous detrimental impact on human life. Any stage of maize vegetative and reproductive growth, such as germination, plant establishment, and flowering is threaten by globally water shortage (Lobell et al., 2014). The emergence rate, uniformity, and robustness of seedlings are crucial factors in determining the yield potential of maize, as they are directly influenced by the initial stages of maize development, namely seed germination and seedling establishment(Tian et al., 2014). However, in arid and semi-arid areas such as northern China, the sowing time of spring maize often encounters serious drought stress, which reduces the emergence rate, hinders the growth of seedlings and usually decreases the grain yield although sometimes the short and mild drought effect may be irreversible. Thus, improving the drought tolerance of maize during its early growth stages is crucial in enhancing both seedling establishment and subsequent growth.

The drought-resistant varieties of maize have a higher, more stable grain yield than drought-susceptible varieties do in both arid, semi-arid zones, and occasional drought areas because of the erratic rainfall. Thus, if the drought-resistant varieties are widely planted in the drought areas, the yield loss caused by drought can be mitigated to a large extent. The drought resistance of crops is a complicated quantitative trait that is controlled by multiple genes (Wang et al., 2019). In the traditional breeding, breeders select drought resistant lines only depending on their performance under drought stress. The characteristics of drought-resistant varieties bred are limited by the drought circumstances under which breeders select the varieties. The breeders will inevitably miss some drought associated genes that are not expressed under those drought circumstances. As a result, the breeders are not able to mine the drought resistance potential maize and to meet the requirements of drought-resistant varieties of maize. Dissecting the genetic structure of drought resistance and identifying the molecular markers will underlie the genetic modification of drought resistance in maize.

Genome-wide association study (GWAS) is an important foundation of molecular marker-assisted breeding technology, which can locate molecular markers related to plant target traits, thereby obtaining quantitative trait locus (QTL) available for breeding selection or for inferring candidate genes to analyze the genetic basis of complex quantitative traits (Aranzana et al., 2005; Wang et al., 2019; Liu et al., 2021b; Ren et al., 2021). Compared with traditional QTL mapping, GWAS has multiple advantages in identifying the genetic architecture of complex traits, which avoids the difficulty of screening large biparental mapping population (Dinka et al., 2007) and can verify gene function by fine mapping and cloning. For instance, a GWAS exploration, using the survival rate and high-quality SNPs of the associated population consisted of 367 maize lines, obtained genetic variations and identified candidate genes, of which the natural variation in *ZmVPP1* (encoding a vacuolar-type H^+^ pyrophosphatase) contributes most significantly to the survival rate, and transgenic maize plants with *ZmVPP1* gene exhibited stronger drought tolerance (Wang et al., 2016). Yet the molecular mechanism of drought resistance of maize remains largely unknown.

The maize plant drought resistance is a quantitative trait with complicated phenotypes, which is difficult to be evaluated. The most evaluations of maize seedlings are usually conducted under osmotic stress caused by PEG solvents or simulated drought by controlling soil water content in the indoor cultivation pool. In this circumstance, the physiological indices such as survival rate and root correlated traits are commonly used to identify the drought resistance. The survival rate refers to the ability of plants to maintain their viability under drought stress and restore normal growth after they obtain sufficient water again. For example, Qin Feng’s team (Liu et al., 2013; Mao et al., 2015; Wang et al., 2016) transferred the three-leaf stage maize seedlings in the cultivation pool without water for drought treatment. When all the seedlings were severely withered, they were rehydrated for a week then to be investigated the survival rates. The team found the survival rates of maize from different genetic resources manifesting a great genetic diversity under drought stress (Wang et al., 2016). Root traits such as root length, total root number, and root surface area can reflect the ability of plants to absorb water and nutrients under drought condition (Guo et al., 2020a). But such physiological indices are not suitable to measure in high throughput in the field condition. Leaf rolling is one of the main responses of maize plants to drought stress which can be scored visually in the field. However, the leaf scoring technique does not meet the high-throughput requirements for efficient phenotypic determination by breeders (Baret et al., 2018). The grain yield (GY) of maize under water shortage is an ultimate index to evaluate its drought tolerance. But, if only depending on this index to research the drought resistance, we are not able to gain insight into the physiological changes that result in the yield declination under drought stress. When maize plants are subjected to drought stress during flowering stage, the development of female flower organs is inhibited more severely than that of male flower organs, and the silking time is delayed and the interval between anthesis and silking is enlarged, which leads to the pistil pollination failure and serious yield loss (Setter et al., 2010). Therefore, the anthesis -silking interval (ASI) is a desired method to evaluate the maize drought resistance under the field condition because it is yield-related, high throughput and cost-effective (Thirunavukkarasu et al., 2014; Farfan et al., 2015). However, so far there is no any index perfectly suitable to evaluate maize drought resistance at seedling stage under the field condition.

An ideal sequencing platform should be high throughput, high genome coverage, high repeatability, and low cost. Among various platforms, genotyping-by-sequencing (GBS) is one of the most extensively used sequencing technologies (Elshire et al., 2011; Guo et al., 2020b; Wang et al., 2020). DArT (Diversity Arrays Technology sequence) is a novel molecular marker identification technology based on gene chip (Kilian et al., 2012), and being used in molecular assisted selection of plants (dos Santos et al., 2016), however, its reliability in maize needs more exploration.

In this study, a maize association panel (NCCP) containing 379 inbred lines were planted in the arid region for 2 years. SNPs genotyped by GBS and DArT, and the combination of SNPs from GBS and DArT were used to conduct GWAS for seedling emergence rate (ER), seedling plant height (SPH) and grain yield (GY) under natural drought conditions. Fifteen common independent and significant SNPs were obtained by BLINK and MLM models, and 15 corresponding candidate genes were identified. The expressions of most of them in the reference inbred line B73 of maize were significantly responded to drought treatment.

## 2 Results

### 2.1 Phenotypic variation

**Figure 1:**
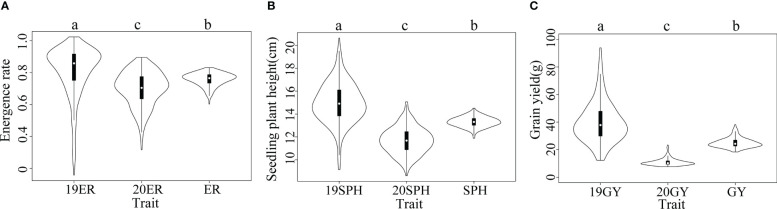
Violin plots of distributions of drought resistance related traits in maize seedlings. The horizontal axis represents different traits. In a violin plot, the inner black box represents the interquartile range. The central white dot represents the median value. The outer white shape on each side represents all measured data points and the thickness represents the probability density of the data. Analysis of variance (ANOVA) was applied to examine the difference of phenotypes among different environments. Different letters indicate statistically significant differences at P ≤ 0.05. **(A)** 19ER, seedling emergence rate measured in Fuxin in 2019; 20ER, seedling emergence rate measured in Fuxin in 2020; ER, seedling emergence rate BLUE value calculated from two-year data; **(B)** 19SPH, seedling plant height measured in Fuxin in 2019; 20SPH, seedling plant height measured in Fuxin in 2020; SPH, BLUE value of seedling plant height calculated from two-year data; **(C)** 19GY, grain yield measured in Fuxin in 2019; 20GY, grain yield measured in Fuxin in 2020; GY, grain yield BLUE value calculated from two-year data.

According to our analysis, the drought resistance related traits in maize seedling in the NCCP panel exhibited extensive phenotypic variation. Normal distributions were observed for the 19SPH, 20SPH and SPH (from the across environment analysis); other traits, 19ER, 20ER, ER (from the across environment analysis), 19GY, 20GY and GY (from the across environment analysis) displayed slightly skewed normal distribution, which are consistent with the performance of quantitative traits (**Supplementary Figure 1**). When the phenotypic variances of traits in different environments were analyzed independently, significant differences were detected in emergence rate, seedling plant height and grain yield among the two environments (**Figure 1**). The median of 19ER was 0.86 and significantly higher than that of 20ER (0.70) and ER (0.77), and the variation range of the emergence rate in 2019 was also broader than that in 2020. The same trend was observed for plant height at seedling stage and grain yield at harvest (**Figure 1**). The phenotypic correlation between BLUE values of the three traits across two environments were positive and significant, as previously reported (Zhang et al., 2022). The results showed that yield trait could be used as the related trait of drought resistance in maize seedling stage, and could reflect the final impact of drought on maize seedling stage.

**Figure 2:**
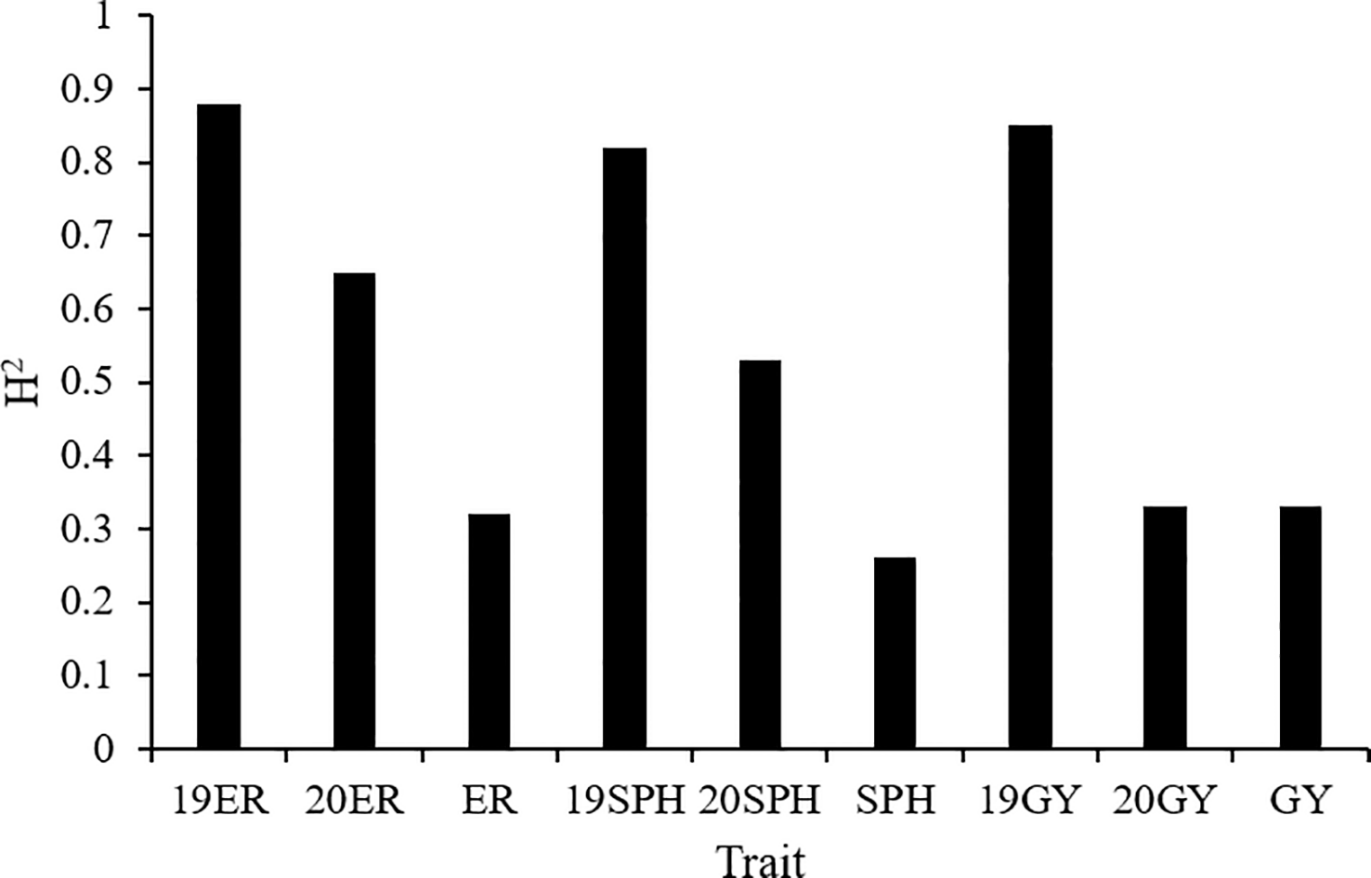
The broad-sense heritability (H^2^) of drought resistance related traits in maize seedlings. 19ER, seedling emergence rate measured in Fuxin in 2019; 20ER, seedling emergence rate measured in Fuxin in 2020; ER, seedling emergence rate BLUE value calculated from two-year data; 19SPH, seedling plant height measured in Fuxin in 2019; 20SPH, seedling plant height measured in Fuxin in 2020; SPH, BLUE value of seedling plant height calculated from two-year data; 19GY, grain yield measured in Fuxin in 2019; 20GY, grain yield measured in Fuxin in 2020; GY, grain yield BLUE value calculated from two-year data.

The broad-sense heritability (H^2^) of the emergence rate ranged from 0.32 in the across environment analysis to 0.88 in the 2019 environment analysis. H^2^ of plant height traits at seedling stage ranged from 0.26 in the across environment analysis to 0.82 in the 2019 environment analysis. For grain yield, H^2^ was 0.85 in the 2019 environment analysis, and 0.33 in both the 2020 environment analysis and across environment analysis (**Figure 2**). The analysis of variance on drought resistance-related traits in maize seedlings showed that genotypic variance had a significant effect (P< 0.01) in all analyses. Additionally, the interaction between genotype and environment (G × E) and environmental variance were also highly significant (P< 0.01) across all environments analyzed (Zhang et al., 2022).

### 2.2 Population structure, kinship, and linkage disequilibrium (LD)

**Figure 3:**
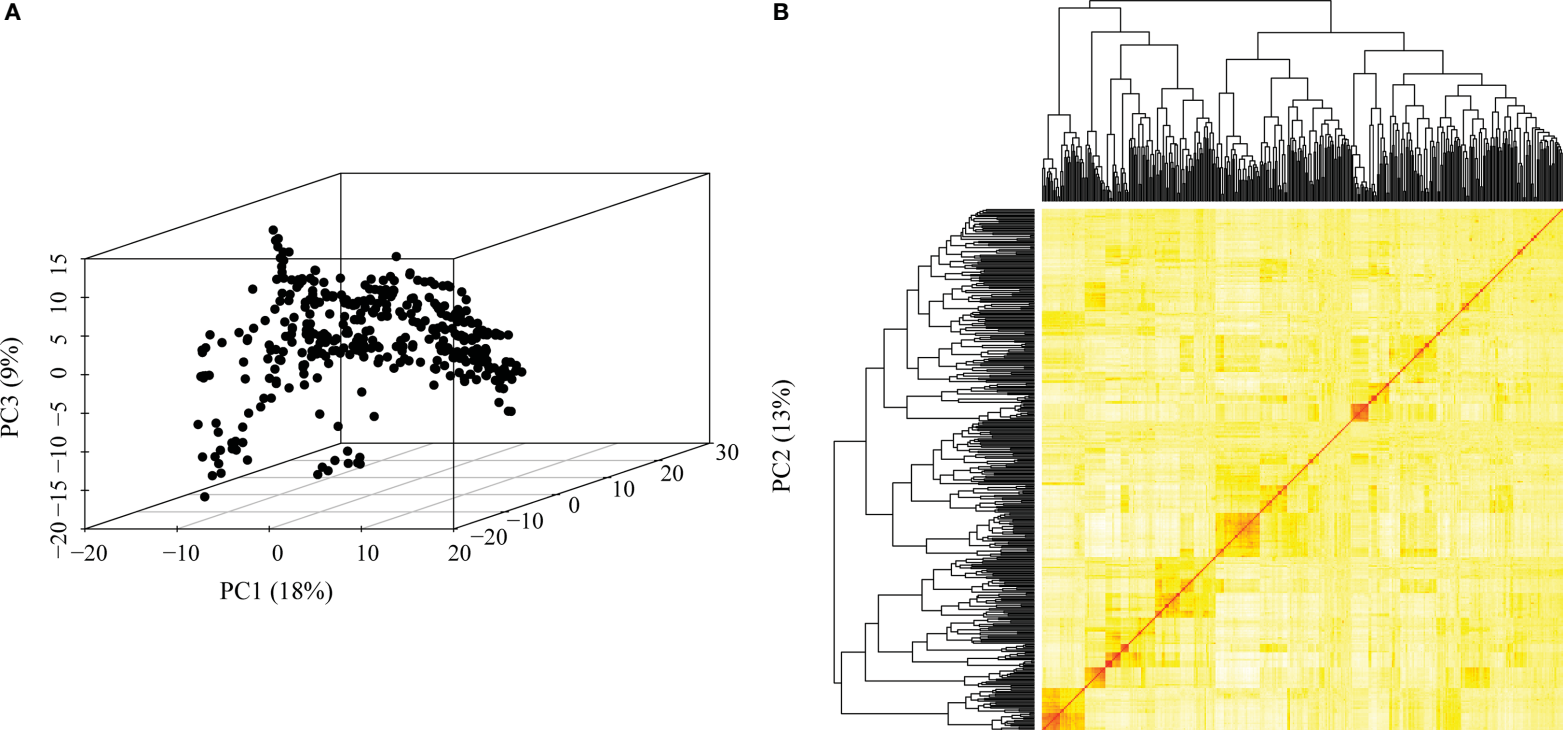
Genetic relatedness among the inbred lines visualized using the heat map, dendrogram of kinship matrix and principal component analysis (PCA) in the association panel. **(A)** Principal component analysis (PCA) in the association population using GBS-DArT dataset, including the first three principal components. The percentage of variation among lines explained by each principal component is displayed on both the x- (PC1), y- (PC2) and z-(PC3) axes. Points in the PCA plot denote individual line; **(B)** The lower right corner is the genetic distance heat map of paired lines, and the upper and left are the dendrogram of kinship matrix in the association panel using GBS-DArT dataset.

We obtained high-quality datasets after filtering, and the average missing rates were 0.073, 0.069, and 0.070 for SNPs from DArT, GBS and the combination of GBS and DArT (GBS - DArT) (**Table 1**). For inbred lines, heterozygosity was also an important index for marker filtering. In this research, the proportion heterozygous of the three marker datasets were less than 0.02 after filtering, which means our material was homozygous and implies the sequencing was accurate (**Figure 3A**). The principal component analysis using 97,862 SNPs of GBS-DArT revealed that there were significant differences among some inbred lines in the association population, and the panel had a strong population stratification structure. Meanwhile, the first three principal components explained 40% of the phenotypic variation rate (**Figure 3A**). A high genetic correlation among inbred lines in the population were shown in the heat map (**Figure 3B**).

**Table 1:** The number of materials and sites, proportion heterozygous and MAF for markers of DArT, GBS and GBS-DArT (combination of both) datasets after filtering.

	DArT	GBS	GBS_DArT
Number of Taxa	379	378	378
Number of Sites	7837	91003	97862
Proportion Missing	0.073	0.069	0.070
Proportion Heterozygous	0.020	0.018	0.018
Average Minor Allele Frequency	0.24	0.233	0.233

**Figure 4:**
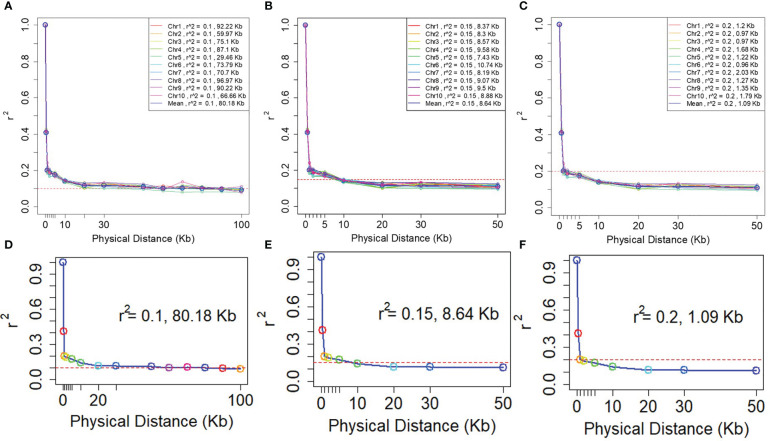
LD decay plots of the NCCP panel. The horizontal axe is the decay distance (Kb) of LD with different r^2^. **(A–C)** are the LD decay for each chromosome; **(D–F)** are the average LD decay for all 10 chromosomes.

A rapid LD decay pattern in the entire panel was observed. The LD decay distance across the 10 chromosomes ranged from 29.46 Kb (Chr5) to 96.67 Kb (Chr8), and the average LD decay distance was 80.18 Kb at an r^2^ value of 0.1. The LD decay distance of 10 chromosomes ranged from 7.43Kb (Chr5) - 10.74Kb (Chr6), the average LD decay distance was 8.61Kb while r^2^ = 0.15. When r2 = 0.2, the LD decay distance of 10 chromosomes was from 0.96Kb (Chr6) to 2.03Kb (Chr7), and the average LD decay distance was 1.09Kb (**Figure 4**). To ensure the accuracy of mapping, it is assumed that at least one SNP marker is in linkage disequilibrium with the QTL of the controlled trait. The minimum number of markers required for GWAS (minimum number of SNP markers = genome size/LD decay distance) are 27,438, 254,629 and 2,018,349 respectively when r^2^ = 0.1, 0.15 and 0.2. Consequently, to assure that the markers have sufficient coverage in the whole genome, the LD decay distance of r^2^ = 0.1 was selected in this study.

### 2.3 Genome-wide association analysis

**Figure 5:**
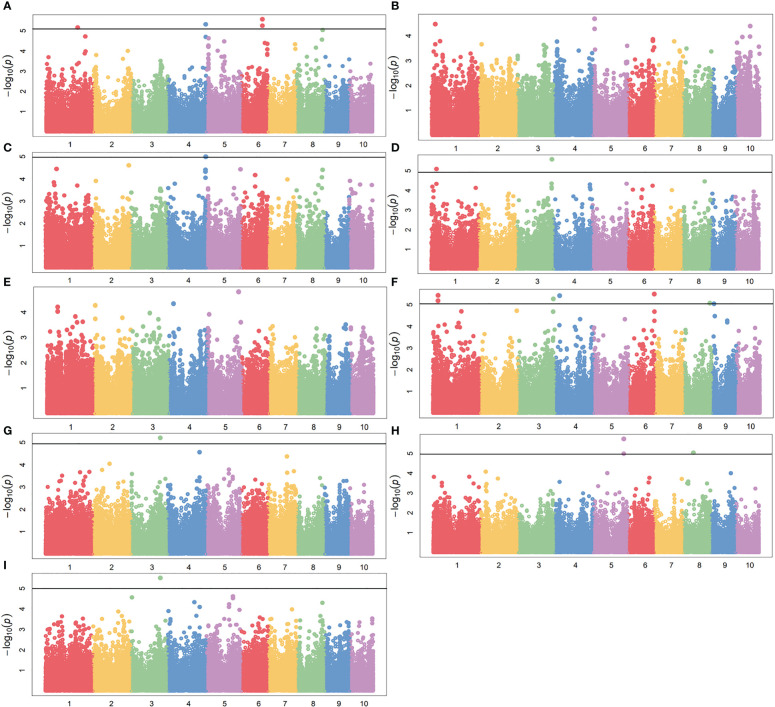
GWAS-derived Manhattan plots showing significant SNPs associated with maize drought resistance traits using MLM. Each dot represents a SNP. The horizontal dashed black line represents the Bonferroni-corrected significant threshold of 1.02 × 10^-5^. **(A)** 19ER: seedling emergence rate (ER) was measured in 19FX (2019 Fuxin); **(B)** 20ER: seedling emergence rate (ER) was measured in 20FX (2020 Fuxin); **(C)** ER: BLUE of seedling emergence rate across two environments; **(D)** 19SPH: seedling plant height (SPH) was measured in 19FX (2019 Fuxin); **(E)** 20SPH: seedling plant height (SPH) was measured in 20FX (2020 Fuxin); **(F)** SPH: BLUE of seedling plant height across two environments; **(G)** 19GY: grain yield (GY) was measured in 19FX (2019 Fuxin); **(H)** 20GY: grain yield (GY) was measured in 20FX (2020 Fuxin); **(I)** GY: BLUE of grain yield across two environments.

**Figure 6:**
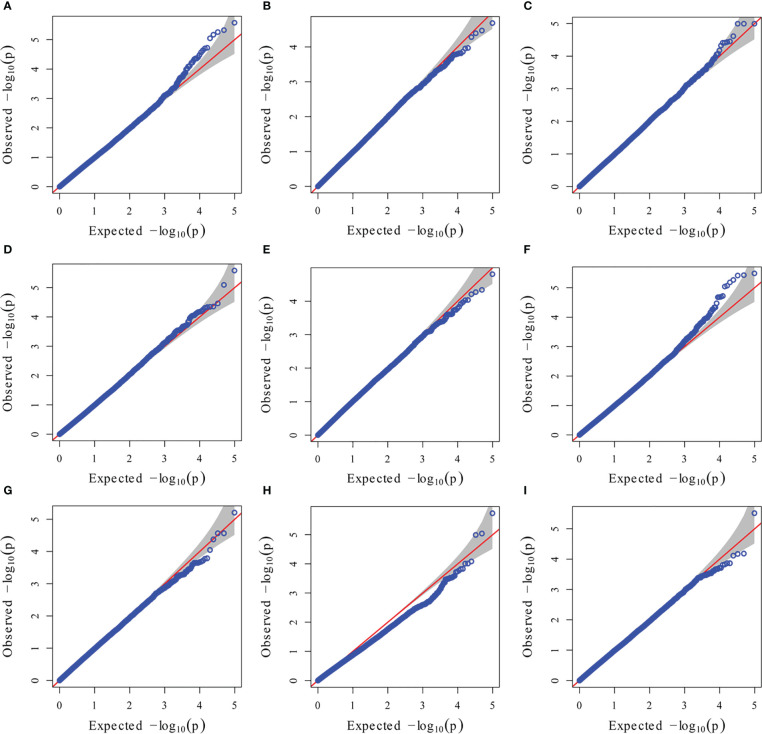
GWAS-derived QQ plots using MLM. **(A)** 19ER: seedling emergence rate (ER) was measured in 19FX (2019 Fuxin); **(B)** 20ER: seedling emergence rate (ER) was measured in 20FX (2020 Fuxin); **(C)** ER: BLUE of seedling emergence rate across two environments; **(D)** 19SPH: seedling plant height (SPH) was measured in 19FX (2019 Fuxin); **(E)** 20SPH: seedling plant height (SPH) was measured in 20FX (2020 Fuxin); **(F)** SPH: BLUE of seedling plant height across two environments; **(G)** 19GY: grain yield (GY) was measured in 19FX (2019 Fuxin); **(H)** 20GY: grain yield (GY) was measured in 20FX (2020 Fuxin); **(I)** GY: BLUE of grain yield across two environments.

To reduce the impact of environmental variability, phenotypic BLUE values across two environments (19FX, and 20FX) were also used for association study. The GWAS analysis was performed using 97,862 SNPs from the combination of GBS and DArT, with a threshold of P< 1.02 × 10^-5^, and the effects of population structure and kinship were considered. A total of 20 and 15 independently significant SNPs were found by applying BLINK (**Supplementary Figures 2**, **3** and **Supplementary Table 1**) and MLM models (**Figures 5**, **6** and **Table 2**), respectively. Among them, 15 SNPs obtained by the two methods are common (**Table 2**). This result indicates that these common SNPs have high reliability. Therefore, the subsequent analysis mainly focused on these 15 common SNPs.

**Table 2:** SNPs significantly associated with drought resistance related traits in maize seedling identified by GWAS using the MLM method.

Trait^a^	SNP	Chr	Pos(bp)	Allele	MAF^b^	P-value	R^2^(%)^c^
19ER	Marker.553171	6	136921236	G/A	0.05	2.66×10^-6^	6.2
	2387359-54-T	4	242397378	C/T	0.27	4.75×10^-6^	5.9
	101239269-43-G	1	204458682	G/A	0.07	6.80×10^-6^	5.7
	2405469-50-C	8	169559968	T/C	0.15	9.01×10^-6^	5.5
ER	Marker.425514	4	244195583	C/A	0.1	1.01×10^-5^	7.0
19SPH	2436908-21-A	3	224392713	G/A	0.06	2.61×10^-6^	6.2
SPH	2432311-16-G	6	167215240	G/T	0.3	3.25×10^-6^	7.5
	2461151-33-G	1	34541393	G/A	0.32	3.73×10^-6^	7.4
	2450059-7-C	4	29282468	C/T	0.14	3.85×10^-6^	7.4
	2428524-68-C	1	34223477	C/T	0.29	6.59×10^-6^	7.0
	2484848-55-T	8	168277928	G/T	0.15	8.53×10^-6^	6.8
	2427986-14-T	9	13708582	T/A	0.11	9.13×10^-6^	6.8
20GY	2428947-36-C	5	197541412	C/A	0.25	1.84×10^-6^	8.7
	2506549-24-A	8	67043226	A/G	0.05	9.12×10^-6^	7.5
19GY/GY	2394080-64-C	3	187093184	C/G	0.15	3.07×10^-6^	7.5

a 19ER, seedling emergence rate measured in Fuxin in 2019; ER, seedling emergence rate BLUE value calculated from two-year data; 19SPH, seedling plant height measured in Fuxin in 2019; SPH, BLUE value of seedling plant height calculated from two-year data; 19GY, grain yield measured in Fuxin in 2019; 20GY, grain yield measured in Fuxin in 2020; GY, grain yield BLUE value calculated from two-year data.

b Minor Allele Frequency.

c Percentage of phenotypic variation explained by the additive effect of the single significant SNP.

For the emergence rate, there were five independent significant SNPs on chromosomes 1, 4, 6 and 8 above the threshold, accounting for 5.5-7.0% of the phenotypic variation, respectively (**Figures 5A–C** and **Table 1**). For the plant height trait at seedling stage, seven independent significant SNPs on chromosomes 1, 3, 4, 6, 8 and 9 above the threshold, accounting for 6.2-7.5% of the phenotypic variation respectively (**Figures 5D–F** and **Table 2**). For the grain yield trait, three independent significant SNPs on chromosomes 3, 5 and 8 above the threshold had been discovered, accounting for 7.5-8.7% of the phenotypic variation, respectively (**Figures 5G–I** and **Table 2**). For these traits, the most significant SNP (2428947-36-C) from 20GY showed the largest phenotypic variation and higher reliability, which signified that the drought resistance in maize seedling stage was a complex quantitative trait controlled by multiple genes with minor effects.

### 2.4 Genotype effects of significant SNPs associated with drought resistance traits in maize seedlings

The allele effects of these 15 overlapping significant SNPs were also evaluated (**Supplementary Figure 4**). The phenotypic distribution differences for different traits between the major alleles and minor alleles were extremely significant for all 15 significant SNPs (P< 0.01). The allele effect of 2394080-64-C on chromosome 3 was the most significant for 19GY/GY phenotypic variation with P-value of 4.4×10^-11^ and 1.1×10^-13^, which was consistent in MLM and BLINK methods (**Supplementary Figure 4**). This indicates that the significant locus identified by GWAS related to drought resistance traits in maize seedlings are reliable and can be utilized for further candidate genes determination. Through the above analysis, the drought susceptible and tolerant alleles of 15 independently significant SNPs were identified. Sequentially, the phenotypic values of 9 different scenarios were sorted from large to small, and the first 20 lines were selected to take the intersection, and 5 lines with a high degree of coincidence were discovered. As shown in **Table 3**, the distribution ratio of tolerant genes was 53.3%-86.7%, which indicates that the more tolerant genes contained in a maize inbred line, the stronger the resistance to drought.

**Table 3:** The drought tolerant alleles and genotype composition of drought-resistant lines of independently significant SNPs.

SNP	drought tolerant allele	NCCP037	NCCP228	NCCP305	NCCP310	NCCP327
Marker.553171	G	G	G	G	G	G
2387359-54-T	C	Y	C	T	C	C
101239269-43-G	G	G	G	G	G	G
2405469-50-C	T	T	T	T	T	T
Marker.425514	C	C	C	C	C	C
2436908-21-A	A	G	G	A	G	G
2432311-16-G	G	G	G	G	G	G
2461151-33-G	A	R	A	A	A	A
2450059-7-C	T	C	C	C	T	C
2428524-68-C	T	Y	T	T	T	T
2484848-55-T	T	K	G	G	T	G
2427986-14-T	T	T	T	T	T	T
2428947-36-C	A	A	A	C	A	A
2506549-24-A	G	A	A	A	A	A
2394080-64-C	C	C	C	C	C	C

The relationship between these significant SNPs and drought resistance in maize seedlings was further verified by searching for candidate genes at significant SNPs and confirming the gene function. During maize breeding, if breeders know drought-resistant SNPs, they can select drought resistant inbred lines or cross combinations and exclude ones without marker SNPs *via* molecular detection. This approach does not require field planting the breeding materials for identifying drought resistance, thus saving a large amount of manpower, land, productive expenses, and time.

### 2.5 Candidate genes selection based on LD of significant SNPs

According to the B73 RefGen v4, nine significant SNPs (Marker.553171, 101239269-43-G, 2405469-50-C, 2432311-16-G, 242852468-C, 2484848-55-T, 2428947-36-C, 2436908-21-A and 2461151-33-G) were just located on the gene sequences, *Zm00001d037771*, *Zm00001d031861*, *Zm00001d012176*, *Zm00001d038930*, *Zm00001d028417*, *Zm00001d012101*, *Zm00001d017495*, *Zm00001d044291* and *Zm00001d028423*, which are directly regarded as candidate genes associated with drought resistance. For the other six significant SNPs, not located on the gene sequence, we perform the linkage disequilibrium (LD) decay within 80Kb (**Supplementary Figure 5**). For 2387359-54-T, 2450059-7-C, 2427986-14-T, 2506549-24-A and 2394080-64-C, the nearest gene is *Zm00001d053859*, *Zm00001d049400*, *Zm00001d045128*, *Zm00001d009488*, *Zm00001d043036*, respectively. In terms of location, the significant SNPs and these candidate genes are within the linkage regions (**Table 4**). For Marker.425514, according to the location and gene function, *Zm00001d053952* located upstream 26,466bp of the SNP was identified as a candidate gene. In this location, the LD decay was larger than 0.6 (**Table 4** and **Supplementary Figure 5**).

**Table 4:** Candidate genes significantly associated with drought resistance related traits in maize seedling identified by GWAS using the MLM method.

Trait^a^	Gene	Chr	Gene interval (bp)	Distance from the related SNP to the edge of the gene (bp)^b^	Annotation	Pathway
19ER	*Zm00001d037771*	6	136915169.136922617	Located	Transcription factor bHLH133	gene expression regulation
	*Zm00001d053859*	4	242396086.242396676	-702	Ethylene-responsive transcription factor 3	gene expression regulation
	*Zm00001d031861*	1	204458557.204459636	Located	Dehydration-responsive element-binding protein 2G	gene expression regulation
	*Zm00001d012176*	8	169557355.169562468	Located	Beta-hexosaminidase	metabolic
ER	*Zm00001d053952*	4	244166895.244169117	-26466	Bax inhibitor 1	programmed cell death
19SPH	*Zm00001d044291*	3	224387800.224393287	Located	Unknown	Unknown
SPH	*Zm00001d038930*	6	167214153.167215654	Located	Transcription factor MYB36	gene expression regulation
	*Zm00001d028423*	1	34540138.34541979	Located	Unknown	Unknown
	*Zm00001d049400*	4	29282763.29284915	295	Transcription factor GTE7	gene expression regulation
	*Zm00001d028417*	1	34216191.34223589	Located	Autophagy-related protein 9	autophagy
	*Zm00001d012101*	8	168277736.168281863	Located	E3 ubiquitin-protein ligase RGLG1	metabolic
	*Zm00001d045128*	9	13703300.13708121	-461	DBP-transcription factor 3	gene expression regulation
20GY	*Zm00001d017495*	5	197539628.197541548	Located	Expansin-B4	cell growth and development
	*Zm00001d009488*	8	67043287.67046926	61	ATP synthase subunit beta	metabolic
19GY/GY	*Zm00001d043036*	3	187095368.187096814	2184	LBD-transcription factor 20	gene expression regulation

a 19ER, seedling emergence rate measured in Fuxin in 2019; ER, seedling emergence rate BLUE value calculated from two-year data; 19SPH, seedling plant height measured in Fuxin in 2019; SPH, BLUE value of seedling plant height calculated from two-year data; 19GY, grain yield measured in Fuxin in 2019; 20GY, grain yield measured in Fuxin in 2020; GY, grain yield BLUE value calculated from two-year data.

b The positive (+) and negative (−) values represent related SNPs location in the 5′ and 3′ direction, respectively, of their candidate gene.

### 2.6 Expression pattern of candidate gene under indoor drought conditions

**Figure 7:**
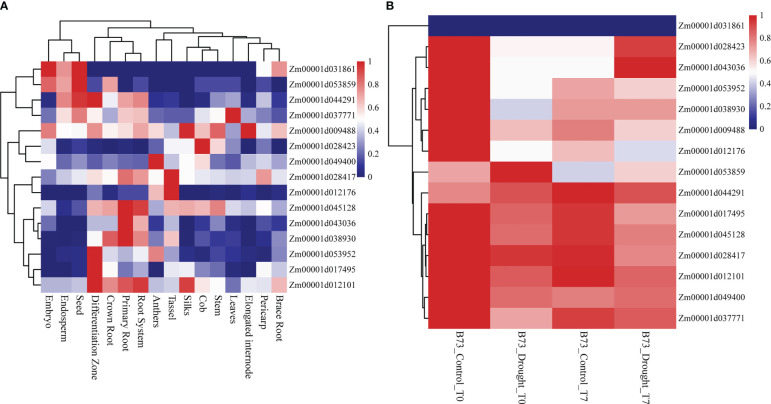
Heat map of expression patterns of candidate genes identified by GWAS in different tissues and different drought treatments. The values used in the figure were normalized TPM value by row. Columns and rows were ordered according to similarity (hierarchical cluster analysis at the top and left. The red and navy blue represented higher and lower expression level. **(A)** Expression of 15 genes in different tissues of maize. **(B)** Expression of 15 genes in different treatments. Drought_T0 is 10 days of drought, Drought_T7 represents 7 days of rehydration after drought and Control is the negative control.

**Figure 8:**
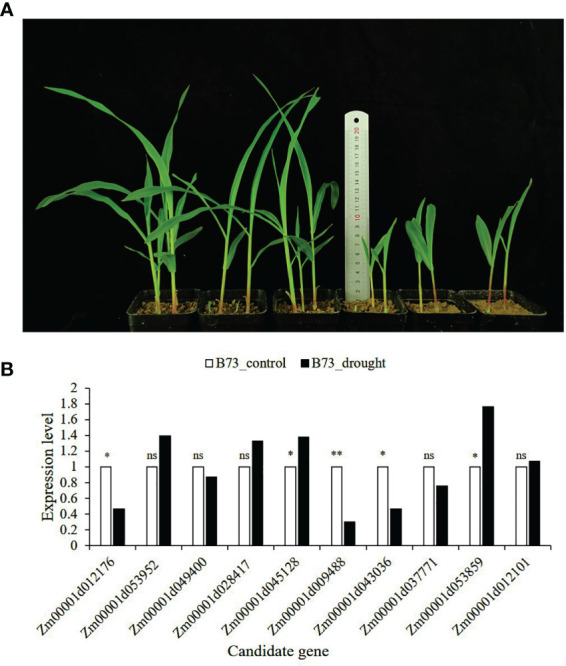
B73 growth plot under normal and drought conditions and the expression of candidate genes verified by qRT-PCR. **(A)** The left side is B73 maize under normal conditions (60% of the maximum water holding capacity), and the right side is B73 maize under drought conditions (30% of the maximum water holding capacity); **(B)** The expression level in the leaves of B73 seedlings under drought stress in laboratory. The value in the figure is 2^-ΔΔCt^. The statistical significance was determined *via* an Independent Samples t-test with **P ≤ 0.01, *P ≤ 0.05, and ns represent not statistically significant (P > 0.05).

All 15 SNPs significantly related to drought resistance traits in maize seedling were identified by GWAS, and 15 candidate genes significantly related to drought resistance traits in maize seedling were obtained (**Table 4**). The 15 candidate genes were divided into five functional types, gene expression regulation, metabolic, programmed cell death, cell growth and development and autophagy (**Table 4**). Tissue specific expression analysis showed that each gene was expressed in different tissues, especially in roots. Interestingly, three genes *Zm00001d017495*, *Zm00001d009488* and *Zm00001d043036* related to yield traits had high expression in differentiation zone, elongated internode and primary root (**Figure 7A**). To further determine whether these genes have functions in drought conditions, their expression patterns were analyzed using published RNA-Seq datasets from control and drought-stressed plants at the V5/V6 developmental stage (Forestan et al., 2016), including 10 days of drought treatment (T0) and 7 days of rehydration (T7) (**Figure 7B**). Compared with Control_T0, one gene is not expressed (*Zm00001d031861*), two are up-regulated (*Zm00001d044291*, *Zm00001d053859*), and the other genes are down regulated under Drought_T0. Compared with Drought_T0, the expressions of *Zm00001d028423*, *Zm00001d043036*, *Zm00001d053952*, *Zm00001d037771*, *Zm00001d038930* were up regulated after 7 days of rehydration and could basically return to Control_T7 level or even higher. Nevertheless, the expressions of the remaining genes were unchanged or even lower, which indicated that drought caused irreversible damage to the activation of these gene expression. In this study, we designed specific primers to verify the expression changes of 10 candidate genes under normal and drought condition at seedling stage (**Supplementary Table 2** and **Figure 8A**). The real-time quantitative PCR indicated that all of 10 genes were sensitive for drought stress and their expression changed under drought conditions, especially *Zm00001d009488* (**Figure 8B**), indicating that these significant SNPs and candidate genes may be potential genetic markers and genes for drought tolerance at seedling stage or in grain yield formation. In this study, we did not detect the change in the expression of the other five candidate genes.

## 3 Discussions

### 3.1 Genetic dissection of drought tolerance in maize seedling

**Figure 9:**
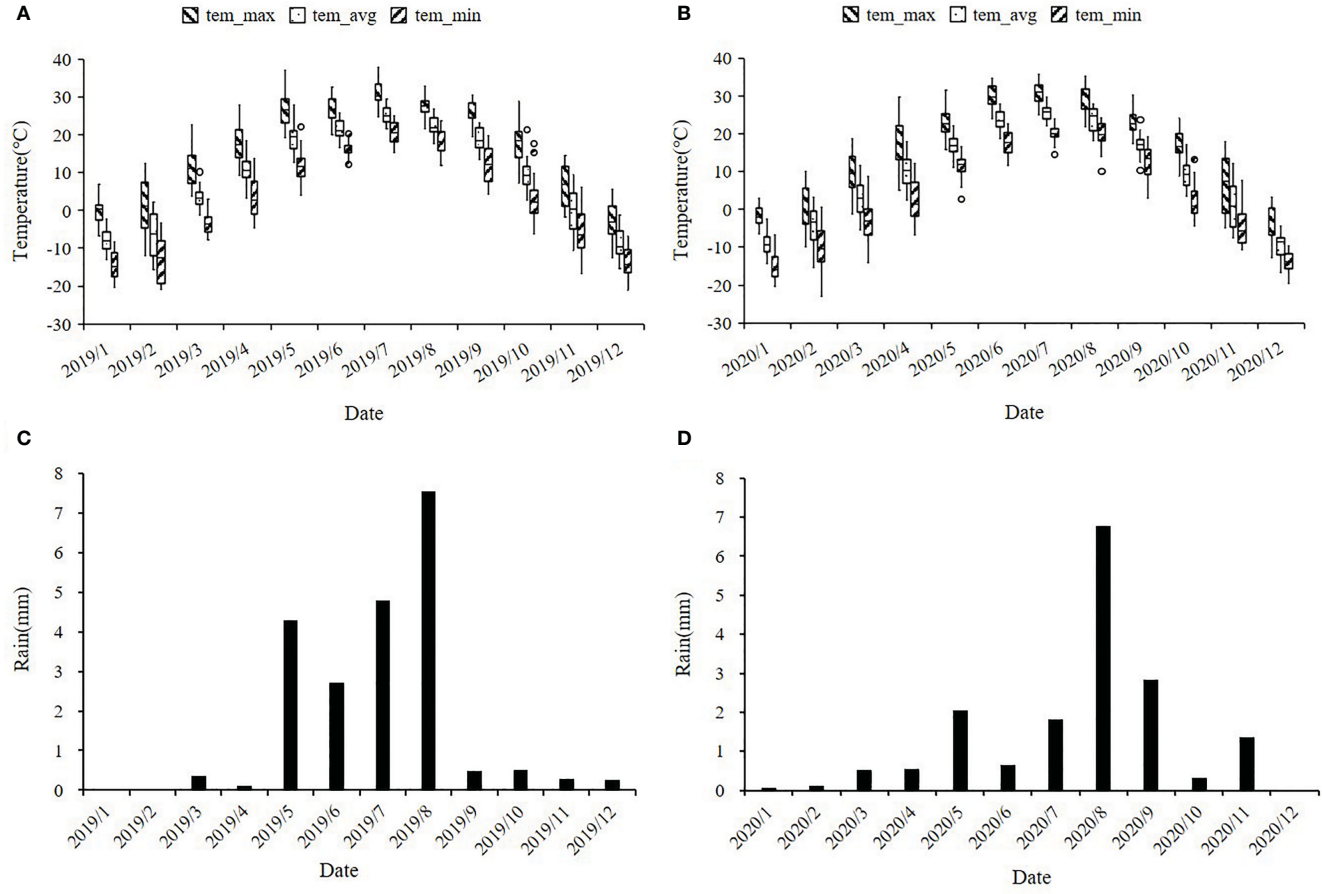
Distribution of temperature and rainfall in maize planting plots of Fuxin Mongolian Autonomous County in 2019 and 2020. **(A, B)** represent the monthly average temperatures (°C) for 2019 and 2020, including the monthly average maximum temperature, average temperature, and minimum temperature; **(C, D)** represent the monthly average rainfall in mm for 2019 and 2020.

Drought tolerance is a complex and inherent characteristic of maize (Wang et al., 2016). Especially in the early development stage, maize seedlings are susceptible to drought stress and the adverse effects generated by severe drought stress on seedling growth are irreversible (Liu and Qin, 2021). Along with rising temperature and dramatically fluctuated rainfall patterns due to the aggravation of the global greenhouse effect in recent years, global maize production has already showed stagnation, especially in arid and semi-arid regions. Fuxin County of Liaoning Province locates in the semi-arid zone of northeast China and the high frequency of spring drought is its typical climate characteristic. In this region, we can conduct larger scale field drought experiments without the use of greenhouses. In our current study, ER, SPH and GY traits exhibited a wide range of phenotypic variations and followed a normal or skewed normal distribution in the NCCP panel (**Figure 1** and **Supplementary Figure 1**). A phenotypic variation analysis uncovered significant differences among different environments, which suggested that different environments could promote or diminish the effects of traits variance. Compared with 2019, 2020 has less precipitation and higher temperature at maize seedling stage, resulting in a smaller phenotypic value (**Figure 9**). According to our genetic analysis, the heritability of ER, SPH and GY were 0.32, 0.26 and 0.33 with a relatively low level, indicating that these traits were controlled by multiple small-effect genes (**Figure 2**). The genotype effects for ER, SPH and GY across two years were significant, indicating the involvement of gene action in the control of drought resistance related traits of maize seedlings (Zhang et al., 2022). The effect of G × E interaction on the traits was highly significant, consistent with the observed phenotypic variation in different years.

### 3.2 Significant SNP involved in drought resistance under field natural drought condition

The two major strategies, linkage mapping and GWAS, have been widely used to identify QTLs for drought resistance in maize, and result in the dissection of many agronomic traits related to drought resistance (Liu and Qin, 2021). In this study, we identified 15 common significant SNPs that contribute to 5.5-8.7% natural variation of drought tolerance in maize seedlings using the MLM and BLINK methods (**Table 2** and **Supplementary Table 1**). There was a different distribution of these SNPs in inbred lines, ranged from 53.3% to 86.7% in 5 drought-resistant inbred lines detected (**Table 3**). For some significant SNPs, although not just located in gene sequences, they all were in the linkage interval (**Table 4** and **Supplementary Figure 5**). These data are consistent with the fact that drought resistance is a complicated trait that may be controlled by multiple quantitative trait loci (QTLs).

### 3.3 Candidate genes and pathways involved in drought resistance under field natural drought condition

At maize seedling stage, the most research focus on the effect of artificial water control or stimulated drought under indoor condition. For instance, the functions of *ZmDREB2.7* (Liu et al., 2013), *ZmNAC111* (Mao et al., 2015) and *ZmVPP1* (Wang et al., 2016) genes in drought resistance have been investigated. However, the mechanism of drought tolerance of maize seedlings in natural field environment is unclear. In this study, the functional annotation revealed that 15 candidate genes could mainly be placed into a few functional groups, such as metabolic, transcriptional regulation, autophagy, cell growth and development, and programmed cell death (**Table 4**). Here, we will discuss how candidate genes may respond to external drought stress.

#### 3.3.1 Transcription factor related candidate genes

Transcription factors (TFs) play an important role in signal transduction networks spanning the perception of a stress signal and the expression of corresponding stress-responsive genes. Multi-gene families (AP2/ERF, bZIP, MYB/MYC, NAC and WRKY) have been discussed in regulating plant drought responses *via* ABA-dependent and/or ABA-independent signaling (Yamaguchi-Shinozaki and Shinozaki, 2006; Hirayama and Shinozaki, 2010; Gahlaut et al., 2016; Hrmova and Hussain, 2021). TFs involved in water stress tolerance in plants may be utilized in future for developing drought-resistant varieties in maize and other crops.

The APETALA2/Ethylene-Responsive Factor (AP2/ERF) gene family constitutes one of the biggest gene families encoding plant-specific transcription factors. This superfamily is characterized by the presence of the AP2/ERF domain, which spans approximately 60 to 70 amino acids and mediates DNA binding. The subfamilies within this superfamily include AP2, RAV, DREB, ERF, and Soloist (Guo et al., 2016; Zhu et al., 2021). The TFs encoded by *Zm00001d031861*, *Zm00001d045128* and *Zm00001d053859* identified in this research belong to the DREB and ERF subfamilies and possess only one AP2/ERF structural domain. The candidate gene *Zm00001d031861* detected in 19ER, encodes dehydration responsive element binding protein 2G, which is a known drought responsive gene at maize seedling stage. Dehydration Responsive Element Binding proteins/C-repeat Binding Factors (DREBs/CBFs, referred as DREBs) are considered to be the major TFs controlling the expression of stress-inducible genes in the ABA-independent pathway (Yamaguchi-Shinozaki and Shinozaki, 2006), and are able to bind a Dehydration Responsive Element (DRE, core motif: A/GCCGAC) in the promoter region of stress-inducible genes (Liu et al., 2013). Two *DREB* genes (*ZmDREB1A* and *ZmDREB2A*) of maize belonging to DREB1 and DREB2 subfamilies, respectively, were cloned and proved to be up-regulated in plants under drought, cold and heat stress (Qin et al., 2004; Qin et al., 2007). Liu (Liu et al., 2013) verified that the DNA polymorphism in the promoter region of *ZmDREB2.7* (*GRMZM2G028386*, B73 RefGen_v3; *Zm00001d031861*, B73 RefGen_v4) is related to drought tolerance of maize seedlings, and overexpression of *ZmDREB2.7* in Arabidopsis and maize showed enhanced tolerance to drought stress. *Zm00001d045128* related to SPH, encodes DBP-transcription factor 3. DNA binding protein phosphatase (DBP) factors are important regulators that participate in both transcriptional regulation and post-translational regulation (Jiao et al., 2020). *ZmDBP3* gene was isolated from maize seedlings and belongs to the A-1 subgroup of the DREBs subfamily. As a trans-acting factor, the ZmDBP3 protein is localized in the nucleus and activates genes containing C-repeat/dehydration-responsive elements (CRT/DRE) under normal growth conditions in transgenic Arabidopsis plants. Overexpression of *ZmDBP3* increased drought and cold resistance in transgenic Arabidopsis (Wang and Dong, 2009). Through transcriptional analysis, the *ZmDBP3* gene was identified as a potential marker for early selection of drought-tolerant maize lines (Marques et al., 2019). *Zm00001d053859*, detected in 19ER, encodes ethylene-responsive transcription factor3 (ERF3), which directly interacts with the GCC box (TAAGAGCCGCC) in the ethylene-responsive element that is necessary and sufficient for the regulation of transcription by ethylene. Transient transfection experiments in tobacco showed that ERFs were localized in the nucleus (Ohta et al., 2000). *Zm00001d053859* may thus regulate drought tolerance in maize by participating in ethylene signal transduction to change the physiological and biochemical reactions of maize seedlings.

The development of lateral roots may be affected by drought. *Zm00001d037771* identified from 19ER encodes bHLH133, pertaining to the basic helix-loop-helix (bHLH) family. The genome-wide analysis identified 208 putative bHLH family proteins in maize (Zhang et al., 2018). A study has indicated that bHLH133 determines the competence of the pericycle for lateral root initiation (Zhang et al., 2021), efficiently obtaining water and nutrients to resist water deficit. Similarly, LBD transcription factor 20 (LBD20) encoded by *Zm00001d043036*, whose Arabidopsis homologous gene *AT2G30340* (Lateral Organ Boundaries Domain 13, LBD13) is a transcription activator located in nuclear, controls the formation of lateral roots in Arabidopsis (Cho et al., 2019).

*Zm00001d038930* encodes the transcription factor MYB36, belonging to the R2R3-MYB subfamily, which plays a central role in controlling plant-specific processes, including primary and secondary metabolism, development, and abiotic and biotic stress responses (Dubos et al., 2010). However, at present, only a few members of the R2R3-MYB family have been well described in maize. By analyzing the expression patterns of 46 *ZmMYB* genes under abiotic stress, researchers found that 13 *ZmMYB* genes responded to drought stress (20% PEG), and the relative expression of *ZmMYB36* was higher at 6 and 12 hours of 20% PEG osmotic stress (Chen et al., 2018). But the mechanism through which this gene can contribute to drought resistance needs further research. The candidate gene *Zm00001d049400* encodes global transcription factor group E7 (GTE7), and Arabidopsis homologous genes *AT5G65630* and *AT5G10550* encode GTE7 and GTE2, respectively. Arabidopsis GTE protein includes plant amphipathic domain (PAD), bromodomain (BRD), extra-terminal domain (ET) and transcriptional activation domains (TAD), which can interact with other TFs. In many cases, BRD can bind to acetylated lysine residues, forming a bridge between acetylated histones and TFs to activate transcription of target genes (Misra et al., 2018). Under drought stress in maize seedling stage, GTE protein may regulate gene expression and signal pathway by interacting with other proteins. But so far, little research has been done on maize GTE protein.

#### 3.3.2 Candidate genes involved in plant cell developmental

The elongation and growth of plant cells will be inhibited under the condition of water scarcity, and the expression of related genes encoding expansins, aquaporins and XETs will also be affected (Wu et al., 2005; Liu et al., 2021a). *Zm00001d017495* identified from 20GY encodes Expansin-B4 (EXPB4). Expansins, driving turgor-driven cell enlargement by unlocking a network of wall polysaccharides (Cosgrove, 2000), are classified into α-expansins (EXPA) and β-expansins (EXPB) (Wu et al., 2001; Cosgrove, 2015). Maize was found to have a total of 88 *ZmEXPs* genes (Zhang et al., 2014). An association study revealed a significantly negative correlation between the level of *ZmEXPA4* expression and anthesis-silking interval (ASI), increasing ASI under drought will lead to low pollination rate and low yield. Furthermore, driving the expression of *ZmEXPA4* using a drought-inducible promoter can significantly reduce ASI under drought conditions (Liu et al., 2021a). Further studies on the function of maize EXPB are still necessary.

#### 3.3.3 Candidate genes involved in cell signal transduction

Many signal molecules, such as intracellular Ca^2+^, ABA, and reactive oxygen species (ROS), are very important for drought signal transduction. One of the first responses of plants to water deficit is the production of ROS. The moderate content of ROS is considered to mediate the induction of defense pathways and help plants adapt to the changing environment (Cruz de Carvalho, 2008). However, if stress continues to aggravate, the increase of ROS will lead to the damage of nucleic acids, proteins, lipids and other cellular components in plants, and causes programmed cell death (PCD) (Çakır and Tumer, 2015). The conserved cell death suppressor Bax inhibitor-1 (BI-1) encoded by *Zm00001d053952* plays a role in the downstream of PCD signaling and inhibits cell death. Studies have indicated that its Arabidopsis homologous gene *AtBI-1* (*AT5G47120*) encodes an endoplasmic reticulum (ER) membrane-associated protein, which modulates ER stress-induced PCD (Ruberti et al., 2018) by interacting with multiple partners to alter intracellular Ca^2+^ flux control (Ishikawa et al., 2011) and fatty acids metabolism (Nagano et al., 2012; Nagano et al., 2019). Simultaneously, the expression of *AtBI-1* cDNA from *Arabidopsis thaliana* in sugarcane, a C4 monocot species, might reduce the activation of cell death pathways initiated by hydric stress or chemical-induced ER stress (Ramiro et al., 2016). Therefore, suppressing the cell death of C4 grasses (including sorghum, maize, and other important crops) is an effective means to improve the long-term drought tolerance of crops. Moreover, ABA signal pathway is also the core of plant response to water deficiency, which has been clearly clarified by the identification of ABA receptors and other signal components. ABA binds to receptor PYRABACTIN RESISTANCE (PYR)/PYR-LIKE (PYL)/REGULATORY COMPONENTS OF ABA RECEPTORS (RCAR), and then the ABA-receptor complex inhibits the activity of clade A type 2 C protein phosphatases (PP2Cs), resulting in the release of SNF1-related protein kinase 2s (SnRK2s) from PP2C-mediated inhibition, further phosphorylation or activation of downstream targets, ABA response transcription factors or ion channels (Danquah et al., 2014; Kumar et al., 2019; Chong et al., 2020; Lin et al., 2021). *Zm00001d012101* encodes E3 ubiquitin-protein ligase RGLG1, and its Arabidopsis homologous genes *AT3G01650* (RGLG1), *AT5G14420* (RGLG2) and *AT1G67800* (RGLG5) have been proved to mediate the signal pathway of drought stress. RGLG1 and RGLG5 interact with PP2Cs, a critical negative regulator of ABA signaling, accelerating PP2CA ubiquitination and degradation under the action of ABA, thereby promoting the activation of the ABA signaling (Wu et al., 2016). Meanwhile, mitogen activated protein kinase 18 (MAPKKK18) may be ubiquitinated at lysine residues K32 and K154 through RGLG1 and RGLG2. The deletion of RGLG1 and RGLG2 can stabilize MAPKKK18 and further enhance the drought resistance of MAPKKK18 overexpressing plants (Yu et al., 2021). Besides, RGLG1 and RGLG2 can also mediate AtERF53 protein degradation by ubiquitination, which has an adverse regulatory effect on drought tolerance (Cheng et al., 2011). Autophagy-related protein 9 (ATG9) encoded by *Zm00001d028417* is the only complete membrane protein in the core mechanism of autophagy and plays an essential role in mediating autophagosome formation (Lai et al., 2020). During a water shortage, autophagy proteins could selectively break down aquaporins to regulate water permeability and damaged proteins to decrease their toxicity. Furthermore, autophagy might also degrade hormone signaling pathway regulators to promote a stress response (Tang and Bassham, 2022).

#### 3.3.4 Candidate genes involved in biosynthesis

*Zm00001d009488* encodes ATP synthase beta subunit, which is associated with the synthesis of ATP in photoreaction to drive carbon assimilation (Shi et al., 2014). Previous studies regarding the expression of ATP-related proteins in response to water deficit are contradictory. According to Tezara et al. (1999) and Valero-Galván et al. (2013), the expression of ATP synthase beta subunit reduces during drought. They suggested that since the cells require less energy during drought, the content of ATPase is likely to decrease in these plants. However, Kottapalli et al (2009); Zhou et al. (2015) and Cao et al. (2017b) observed the opposite, which higher expression of the ATP synthase beta subunit might enhance the energy supply to protect plants from damage under drought stress conditions (Rashid et al., 2022). *Zm00001d012176* encodes beta-hexosaminidase, a homologous gene of Arabidopsis beta-hexosaminidase 3 (hexo3), which can participate in the formation of paucimannosidic N-glycans (Liebminger et al., 2011), and Expression Pattern of Candidate Genes.

The specific high expression of different genes in the primary root and differentiation zone endows maize plants with resistance to water stress (**Figure 7**). At the same time, the three genes (*Zm00001d017495*, *Zm00001d009488* and *Zm00001d043036*) identified for grain yield are also highly expressed in primary tissues, which may play a crucial role in the drought resistance of maize seedlings. It also indicates that grain yield can be used as a drought resistance indicator of maize seedlings, reflecting the ultimate drought resistance of maize plants. In our qRT-PCR experiments, the results are basically consistent with Forestan (Forestan et al., 2016), but *Zm00001d045128* was up-regulated in the leaves of B73 seedlings under drought stress in laboratory, which may be caused by experimental differences (**Figure 8**). In particular, *Zm00001d009488* were down-regulated in the leaves of B73 seedlings under drought stress in laboratory, which is consistent with the results of Tezara (Tezara et al., 1999) and Valero-Galván (Valero-Galván et al., 2013). Kottapalli (Kottapalli et al., 2009) indicated that ATP synthase beta subunits are only highly induced in drought tolerant genotypes, but B73 is drought susceptible genotype.

## 4 Materials and methods

### 4.1 Association mapping panel

379 inbred lines, collected from China, America, Mexico, and other regions, among them, most from Northeast China, regarded as the northeast China core population (NCCP), were utilized to conduct GWAS in the current study. The heterosis group of the NCCP can be divided into 13 groups, including the Jidan, Longdan, NSS, SS, Huanglvxi, Lvxi, Reid, France, PA, Lvdahonggu, Tangsipingtou, Mixed and Unknown. The CML series from CIMMYT are tropical materials, and only two can be grown in Northeast China, here they were classified into the Mixed group. Some small groups and inbred lines from the multiple parent selection were classified as unknown group.

### 4.2 Field planting and phenotypic measurement

All 379 inbred lines of the association panel were planted in the Fuxin Mongolian autonomous county, Liaoning Province, China (42°06′N, 122°55′E) in 2019 (19FX) and 2020 (20FX), where drought occurs frequently and seriously in spring season. The seeds were sown on 12 May 2019 and 11 May 2020, respectively, and harvested on 7 October 2019 and 9 October 2020, respectively. During the 2019 maize growing season, the average temperature was 21°C and the average rainfall was 4 mm. In the 2020 maize growing season, the average temperature was 21°C and the average rainfall was 3 mm. During the maize seedling stage (May and June), maximum temperatures of 35°C across two years and average monthly precipitation of less than 5 mm caused severe drought stress at the seedling stage, maintaining three leaves throughout the growing season (**Figure 9**).

The maize field in our study were not irrigated, but seeds were sown before precipitation according to weather forecasts for germination. A completely randomized block design with three replications was used in each evaluation environment. Inbred lines were planted using one seed per hole on a single plot of 3 m length, with 10 cm spacing between plants and 60 cm spacing between rows. The target traits of seedling emergence (ER), seedling plant height (SPH) at the three-leaf stage and grain yield (GY) at the harvest stage were evaluated to represent the drought tolerance of the tested plant line. ER and SPH were measured at the seedling stage, i.e. 20 days after sowing. ER was measured as the ratio of surviving seedlings to the number of seeds sown. SPH was measured as the distance from the base of the plant to the highest point of the seedling. While GY was measured at the maturity stage, moisture content readings were taken by the LDS-1G Grain Moisture Meter. The average dry weight of five evenly grown ears was considered as the final yield of each inbred line.

### 4.3 Statistical analysis of phenotypes

The best linear unbiased prediction (BLUE) values and broad-sense heritability (H^2^) of ER, SPH, and GY were calculated within and across environments using the META-R software version 6.04 (http://hdl.handle.net/11529/10201). The linear mixed models used in META-R are implemented in the LME4 R-package, functions of lmer () and REML were used to estimate the variance components.


$$Y_{ijk}=\mu +Gen_{i}+Env_{j}+Gen_{i}\times Env_{j}+Rep_{k}+\epsilon_{ijk}$$

Where $Y_{ijk}$ is the trait of interest, μ is the overall mean, $Gen_{i}$ , $Env_{j}$ , and $Gen_{i}\times Env_{j}$ are the effects of the *i-th* genotype, *j-th* year, and *i-th* genotype by *j-th* year interation, respectively. $Rep_{k}$ is the effect of *k-th* replication. $\epsilon_{ijk}$ is the residual effect of the *i-th* genotype, *j-th* year, *k-th* replication. Genotype is considered as the fixed effect, whereas all other terms are declared as the random effects. Years with heritability below 0.05 were excluded from the across environment analysis.

Broad-sense heritability (H^2^) based on the entry means within trials was estimated as follows:


$$H^{2}=\frac{\sigma_{g}^{2}}{\sigma_{g}^{2}+\frac{\sigma_{ge}^{2}}{nEnvs}+\frac{\sigma^{2}}{nEnvs\times nreps}}$$

Where $\sigma_{g}^{2}$ , $\sigma^{2}$ , and $\sigma_{ge}^{2}$ are the genotypic variance, error variance, and genotype-by-environment interaction variance, respectively, and *nreps* and *nEnvs* are the numbers of replications and environments, respectively.

### 4.4 Genotyping and quality control

Leaf samples were collected from each maize inbred line seedling for DNA extraction with a CTAB procedure, and genotyping was carried out by DArT and GBS platform. GBS was performed by the common protocol in maize in Wuhan Huada medical laboratory Co., Ltd (Elshire et al., 2011). The genomic DNA was digested with restriction enzyme *ApeKI*, and a DNA library was constructed in 96-plex and sequenced on Illumina-hiseq2500/4000 platform. Details in single nucleotide polymorphism (SNP) calling and imputation have been previously described (Cao et al., 2017a). Initially, 768,558 SNPs evenly distributed on maize chromosomes were called for each line; 760,831 of them were assigned to chromosomes 1-10, and 7,727 could not be anchored to any of the chromosomes.

DArT was completed in the SAGA sequencing platform established by Mexico DArT company and CIMMYT. It uses two enzymes (*PstI* and *HpaII*) to cut DNA samples to reduce the complexity of genome. After enzyme digestion, DNA from different samples was linked with barcodes of different base combinations and sequenced to construct a DNA simplified sequencing library. Combined with Illumina second-generation short fragment sequencing technology (150bp), the simplified sequencing DNA library of mixed samples was sequenced on a single sequencing lane (https://www.diversityarrays.com/) (Kilian et al., 2012). At first, 39,659 DArT SNPs were called for each line, of which 39112 were located on maize chromosomes 1-10, and 547 SNPs could not be anchored on any maize chromosomes.

In DArT and GBS datasets, TASSEL 5 (Bradbury et al., 2007) was used to filter out markers with minor allele frequency (MAF)< 0.05 and a missing rate > 20%. There were 379 lines and 7,837 markers remaining in DArT datasets, 378 lines and 91,003 SNPs remaining in GBS datasets. The remaining markers of the two sequencing platforms were combined and filtered again. Imputation approaches used the method of Cao (Cao et al., 2017a). Finally, we obtained 97,862 GBS-DArT SNPs of 378 lines for subsequent analysis.

### 4.5 Genome-wide association mapping and phenotypic variance contributions of significant loci

We used the trait values of single environment and BLUE in the Tab separator format and performed the GWAS analysis using a genotype with 97,862 SNP markers from GBS-DArT combination (MAF ≥ 0.05 and missing rate ≤ 20%). The mixed linear model (MLM) of GAPIT (Genomic Association and Prediction Integrated Tool) package was used to perform the association analysis for drought resistance related traits, and it is one of the most effective methods for controlling false positives in GWAS. This model simultaneously incorporates both population structure and kinship (Yu et al., 2006). The MLM model is described as follows:


y=Xβ+Qv+Zu+ϵ

Where X is the SNP marker matrix, Q and Z represent the subpopulation membership matrix and kinship matrix respectively, β and v are the coefficient vectors of SNP markers and subpopulation membership respectively, u is the random genetic effect vector, and ϵ is the random error vector (Yu et al., 2006; Fan et al., 2016).

Simultaneously, association analysis for all the traits was also conducted with the model of Bayesian information criterion and Linkage-disequilibrium Iteratively Nested Keyway (BLINK) (Huang et al., 2019). The BLINK package can be downloaded from https://github.com/YaoZhou89/BLINK. The first three PCs were treated as covariates to perform GWAS. The principal component analysis (PCA) and kinship of 378 maize inbred lines were conducted using prcomp() and GAPIT.kinship.Zhang() function in R(v 4.1.1), separately.

The linkage disequilibrium (LD) of the entire panel was analyzed using TASSEL software (Bradbury et al., 2007), and LD decay plots were done using the LD decay Plot Tool written by Zhang Ao based on the base functions using the R language. The plotting script is available online on the webpage https://aozhangchina.github.io/R/LDdecay/LDdecayPlotTool.html. Then, we used the uniform Bonferroni-corrected threshold of α = 1 for the mixed linear model’s significance cutoff as reported in previous studies (Mao et al., 2015; Liu et al., 2022). Therefore, the suggested P-value was computed with 1/n (n = 97,862, total markers used), and we obtained a P-value threshold of 1.02×10^-5^ for GWAS.

### 4.6 Annotation of candidate genes

The most significant SNP was chosen to represent the locus in the same LD block (r^2^< 0.2). The physical locations of the SNPs were determined in reference to the B73 RefGen_v4. LD, which was calculated for each independent significant SNP within its surrounding regions (80Kb). Candidate genes were identified by the presence of significant SNPs directly located within the gene sequence. Alternatively, genes residing within a corresponding LD region also were considered, and their biological functions were annotated using data from the MaizeGDB and UniProt website.

### 4.7 Heat map of candidate genes

The expression levels of candidate genes from different tissues and different drought treatments of B73 were downloaded from the MaizeGDB qTeller (https://qteller.maizegdb.org/). The values used for heat map construction were calculated as normalized TPM value by row.

### 4.8 RNA isolation and quantitative RT–PCR analysis

B73 seeds were sown in a cultivating pot (upper diameter 6.5cm, lower diameter 4.5cm, height 7.5cm). The test soil was clayey brown soil, collected from the northern experimental station of Shenyang Agricultural University in 2020 and baked at 120°C until the soil weight was no longer changed. Four seeds were sown in each small pot and repeated three times for two treatments. The soil water content of normal control was 60% of the maximum water holding capacity, and that of drought treatment was 30% of the maximum water holding capacity. Total RNA from maize leaves was extracted on the 12^th^ day after sowing.

Total RNA was extracted using SteadyPure Plant RNA Extraction Kit (AG2109S) of Accurate Biotechnology from at least three seedlings of B73. Subsequently, the concentration of RNA was determined by BioDrop. cDNA was prepared using PrimerScriptTM RT reagent Kit with gDNA Eraser (Takara Biotechnology Dalian Co. Ltd.). The maize Ubi1 (UniProtKB/TrEMBL, Q42415) gene was used as an internal control to normalize the data, and the candidate gene primers were synthesized by Sangon Biotech company and purified by PAGE. qRT-PCR was performed on Bio-Rad CFX Connect Real-Time System (CA, USA) with a 10 μl reaction volume containing 4.6 μl of ddH2O, 3.6 μl of TB GreenTM Premix Ex TaqTM (Tli RNaseH Plus; TakKaRa), 0.4 μl of specific primers (10 μM) and 1 μl of cDNA. PCR conditions consisted of an initial denaturation step at 95°C for 40 seconds, followed by 39 cycles at 95°C for 5 seconds and 60°C for 1 minute, and a final stage of 60-95°C to determine melting curves of the amplified products. The quantification method (2^-ΔΔCt^) was used and the variation in expression was estimated using three biological replicates.

## 5 Conclusions

In this study, we have explored the genetic basis of the performance of drought tolerance at seedling stage of maize natural population under natural field condition by applying GWAS approach. Drought resistance related traits manifested a relatively low heritability and a broad variation in the association panel. According to the GWAS, multiple genetic loci with small effects regulate the natural variation in ER, SPH and GY in maize under drought condition. 15 commonly significant SNPs were obtained by the MLM and BLINK method, and the phenotypic distribution of major and minor alleles in different traits showed extremely significant difference (P< 0.01). Simultaneously, the distribution ratio of tolerant genes was 53.3% - 86.7% in 5 drought-resistant inbred lines. We further found 15 candidate genes that may involve in plant cell developmental, cell signal transduction, and transcription factors. These candidate genes provide valuable resources for further investigation to dissect the molecular network to regulate drought resistance of maize seedlings to increase yield. In addition, the significantly associated SNPs found in this research will be helpful in facilitating marker-assisted selection of drought tolerance of maize seedlings in breeding programs.

## Data availability statement

The original contributions presented in the study are publicly available. This data can be found here: https://www.ebi.ac.uk/eva/?eva-study=PRJEB61663.

## Author contributions

AZ and YR initiated and designed the overall study. DD, SJ, CZ, and CL performed and coordinated the field experiments and phenotypic data collection. HZ, YL, XD, and YG carried out the data analysis. SC and AZ interpreted the results and wrote the manuscript. All authors contributed to the article and approved the submitted version.
