# Supplementary material for: Genome-wide association study presents insights into the genetic architecture of drought tolerance in maize seedlings under field water-deficit conditions
Source: Front Plant Sci. 2023 May 8;14:1165582. doi: 10.3389/fpls.2023.1165582 (PMC10200999; doi:10.3389/fpls.2023.1165582)
Supplement: Supplementary file 1 [file Table_1.docx]

Supplementary Material

Genome-Wide Association Study Presents Insights into the Genetic Architecture of Drought Tolerance in Maize Seedlings under Field Water-Deficit Conditions

Shan Chen, Dongdong Dang, Yubo Liu, Shuwen Ji, Hongjian Zheng, Chenghao Zhao, Xiaomei Dong, Cong Li, Yuan Guan, José Crossa, Ao Zhang*, Yanye Ruan*

*** Correspondence:** Ao Zhang, Yanye Ruan: zhangao7@syau.edu.cn, yanyeruan@syau.edu.cn

**Supplementary Tables 1.** SNPs chromosomal positions significantly associated with maize drought resistance traits identified by GWAS using the BLINK method

| Trait^a^ | SNP | Chr | Pos(bp) | Allele | P.value | MAF^b^ |
| --- | --- | --- | --- | --- | --- | --- |
| 19ER | 2387359-54-T | 4 | 242397378 | C/T | 2.77E-06 | 0.27 |
|  | Marker.553171 | 6 | 136921236 | G/A | 2.84E-06 | 0.05 |
|  | 101239269-43-G | 1 | 204458682 | G/A | 9.16E-06 | 0.07 |
|  | 2405469-50-C | 8 | 169559968 | T/C | 9.23E-06 | 0.15 |
| 20ER | 2434311-56-C | 5 | 11958310 | C/G | 8.62E-06 | 0.27 |
| ER | Marker.425514 | 4 | 244195583 | C/A | 5.40E-06 | 0.10 |
|  | Marker.251530 | 2 | 229074097 | A/T | 7.47E-06 | 0.07 |
| 19SPH | 2436908-21-A | 3 | 224392713 | G/A | 1.58E-06 | 0.16 |
| SPH | 2432311-16-G | 6 | 167215240 | G/T | 1.36E-06 | 0.30 |
|  | 2461151-33-G | 1 | 34541393 | G/A | 1.61E-06 | 0.32 |
|  | 2450059-7-C | 4 | 29282468 | C/T | 1.65E-06 | 0.14 |
|  | 2428524-68-C | 1 | 34223477 | C/T | 3.09E-06 | 0.29 |
|  | 2484848-55-T | 8 | 168277928 | G/T | 4.12E-06 | 0.15 |
|  | 2427986-14-T | 9 | 13708582 | T/A | 4.47E-06 | 0.11 |
| 19GY | Marker.609746 | 7 | 121237331 | G/T | 1.05E-09 | 0.17 |
|  | 2434342-32-A | 2 | 57880149 | A/T | 1.09E-09 | 0.29 |
|  | Marker.411465 | 4 | 204030923 | C/G | 2.15E-07 | 0.31 |
|  | 2394080-64-C | 3 | 187093184 | C/G | 3.45E-07 | 0.15 |
| 20GY | 2428947-36-C | 5 | 197541412 | C/A | 7.51E-08 | 0.25 |
|  | 2506549-24-A | 8 | 67043226 | A/G | 6.48E-07 | 0.05 |

^a^19ER, seedling emergence rate measured in Fuxin in 2019; 20ER, seedling emergence rate measured in Fuxin in 2020; ER, seedling emergence rate BLUE value calculated from two-year data; 19SPH, seedling plant height measured in Fuxin in 2019; SPH, BLUE value of seedling plant height calculated from two-year data; 19GY, grain yield measured in Fuxin in 2019; 20GY, grain yield measured in Fuxin in 2020. ^b^Minor Allele Frequency.
