# Supplementary material for: Genome-wide association study presents insights into the genetic architecture of drought tolerance in maize seedlings under field water-deficit conditions
Source: Front Plant Sci. 2023 May 8;14:1165582. doi: 10.3389/fpls.2023.1165582 (PMC10200999; doi:10.3389/fpls.2023.1165582)
Supplement: Supplementary file 2 [file Table_2.docx]

Supplementary Material

Genome-Wide Association Study Presents Insights into the Genetic Architecture of Drought Tolerance in Maize Seedlings under Field Water-Deficit Conditions

Shan Chen, Dongdong Dang, Yubo Liu, Shuwen Ji, Hongjian Zheng, Chenghao Zhao, Xiaomei Dong, Cong Li, Yuan Guan, José Crossa, Ao Zhang*, Yanye Ruan*

*** Correspondence:** Ao Zhang, Yanye Ruan: zhangao7@syau.edu.cn, yanyeruan@syau.edu.cn

**Supplementary Tables 2.** Sequence of the primers used in this study

| Primer name | Forward (5'→3') | Reverse (5'→3') |
| --- | --- | --- |
| Actin | GTGTCCTGTCCACCCACTCTCT | GGAACTCGTTCACATCAACGTTC |
| *Zm00001d037771* | GCAACAGCACCGTAACAGG | TTGCCAAACGGAGAAACGA |
| *Zm00001d053859* | TCCTCGTCGTCGGTCCTC | TCAGATCGAACGGCAACGG |
| *Zm00001d012176* | GTGCGTACACCTGAAGACGA | CAGTGGGTCTCCTGTCGTTG |
| *Zm00001d053952* | ACCTGGGCGGACTGCTAT | CGATGACCTCCTGCGTGT |
| *Zm00001d049400* | CGGCACCGTCAAGCAGAA | TGGTCGGGCGGGTTGTA |
| *Zm00001d028417* | ACTTGAGCCCCTTGGGATTG | GCTCTATCCGAGTGTGGTGG |
| *Zm00001d012101* | CGTGGAATTAGGGATTTTGGG | TGAAGAACTCTGAGGGTAGGTGC |
| *Zm00001d045128* | ATGGCTATCTGAACGGTCTGC | TCGTCAGCGTGGTCATCTTG |
| *Zm00001d009488* | CGACAAACCACTTCCTCCCT | GCACCACCGAATAGACCAAT |
| *Zm00001d043036* | TCCAGCAACAGGTGAACGC | CGCAGGAGCCAAGACGAA |
