# Supplementary figures and images for: Genome-wide association study presents insights into the genetic architecture of drought tolerance in maize seedlings under field water-deficit conditions
Source: Front Plant Sci. 2023 May 8;14:1165582. doi: 10.3389/fpls.2023.1165582 (PMC10200999; doi:10.3389/fpls.2023.1165582)

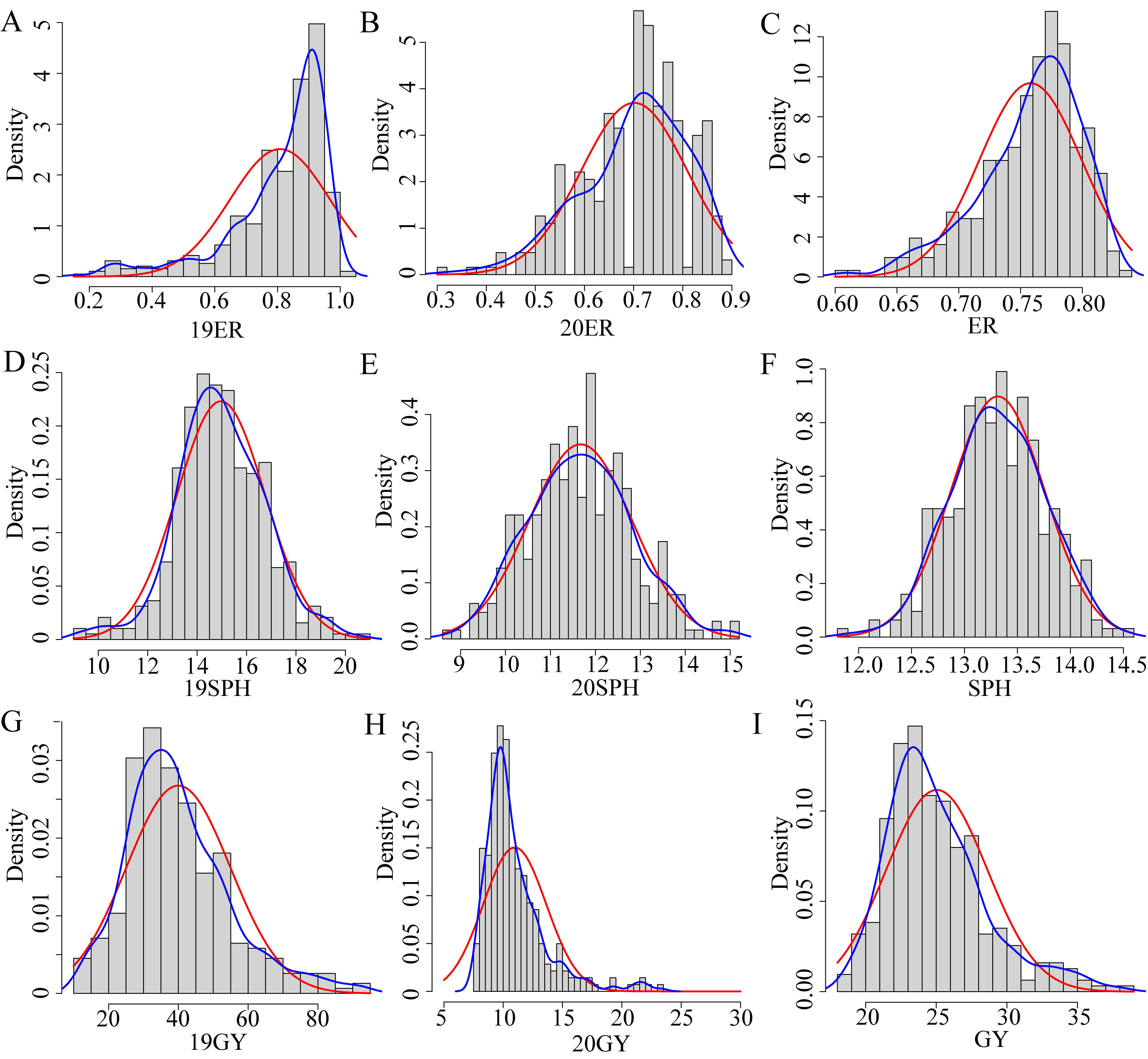

Supplement: Supplementary file 3 [file Image_1.png]

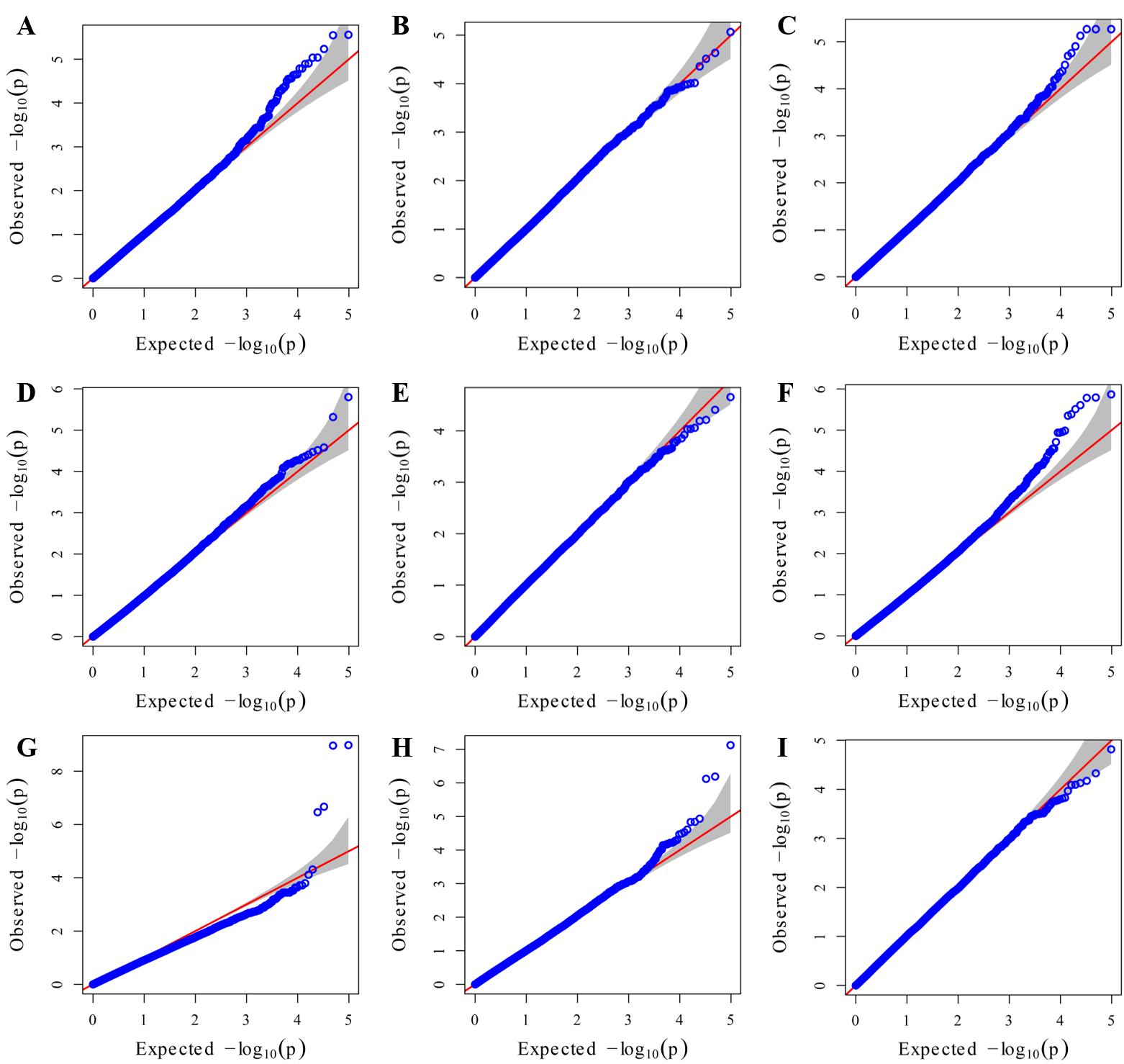

Supplement: Supplementary file 5 [file Image_3.tif]

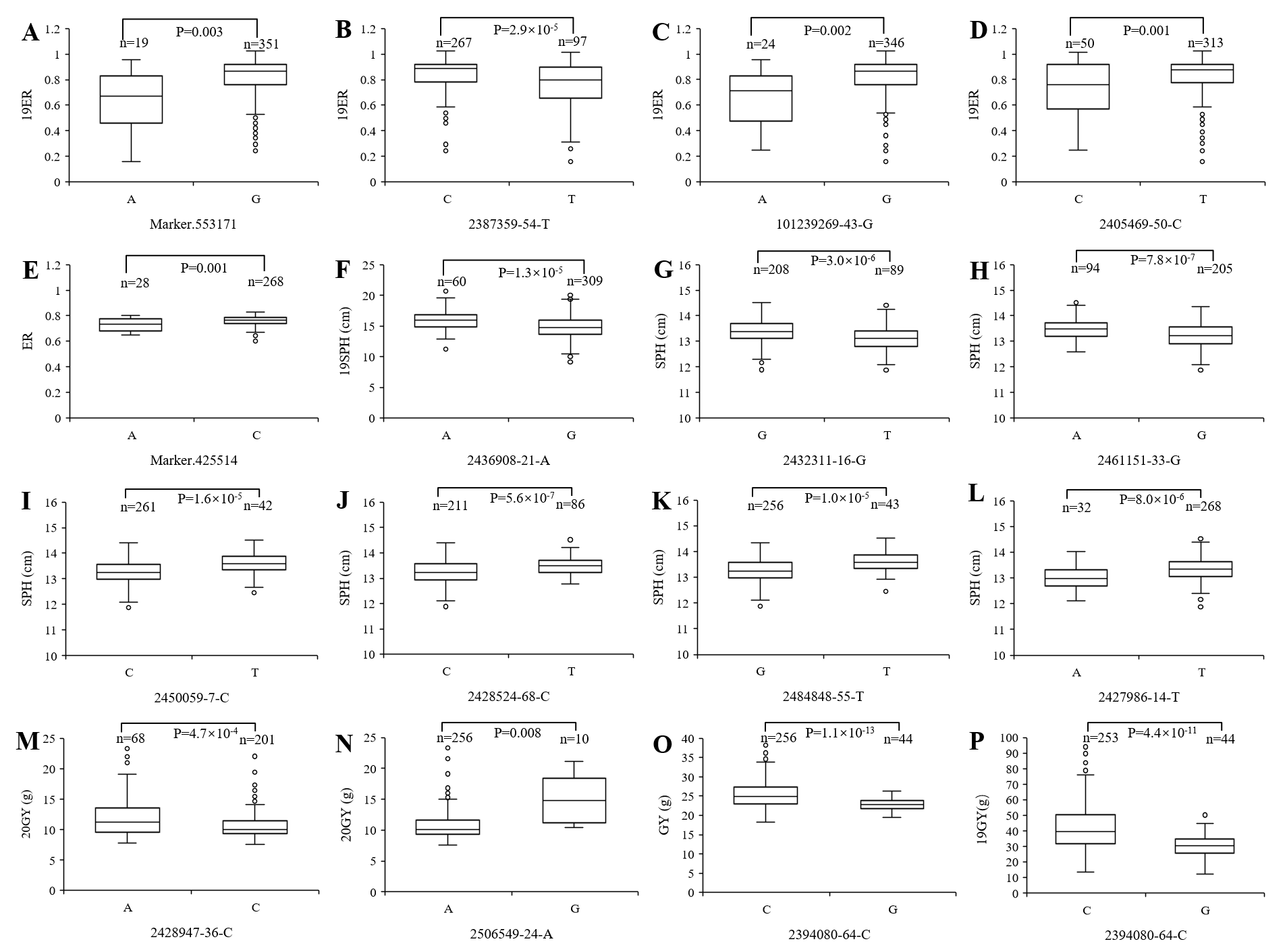

Supplement: Supplementary file 6 [file Image_4.tif]

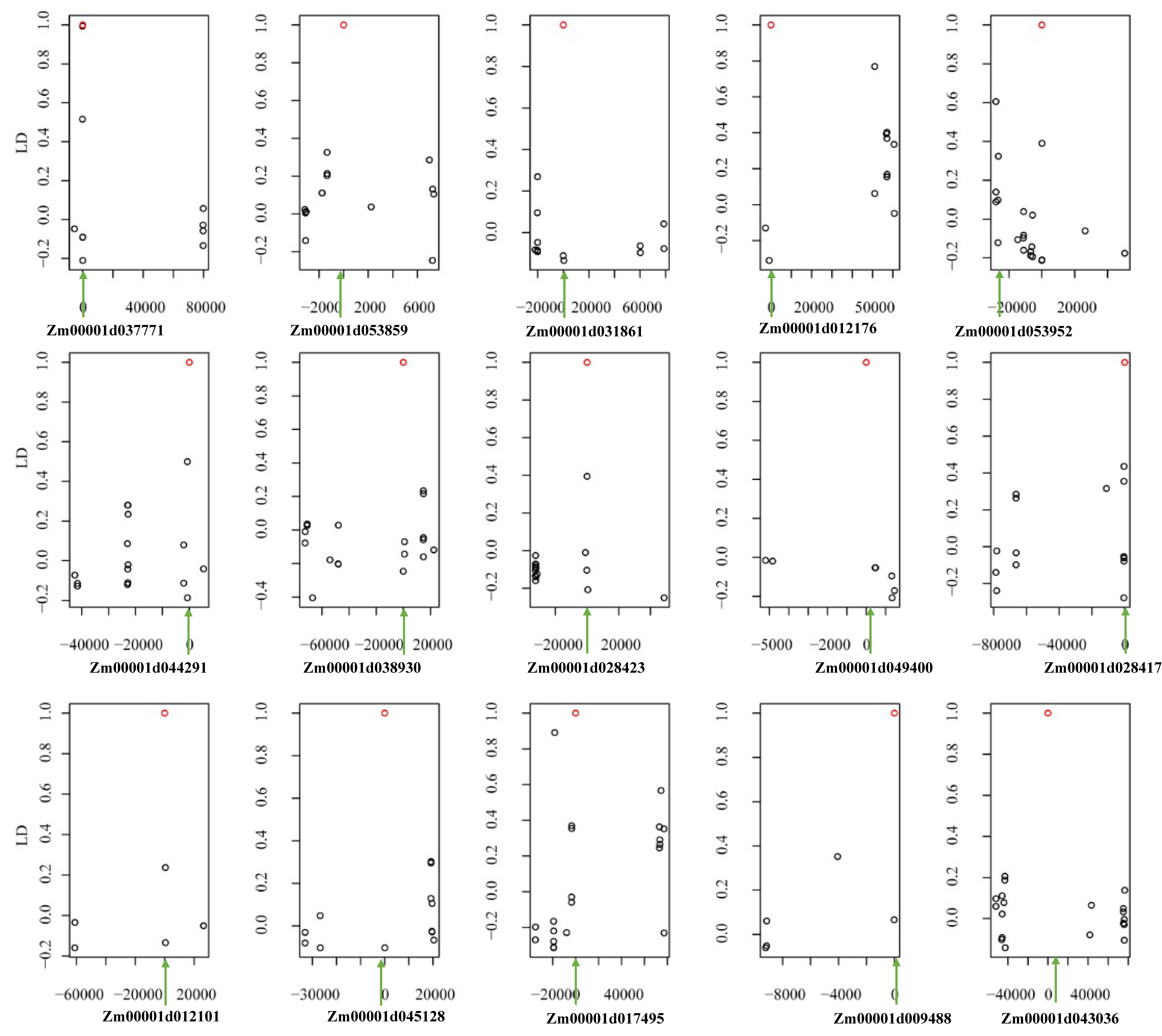

Supplement: Supplementary file 7 [file Image_5.png]
